# The community pharmacy setting for diabetes prevention: Views and perceptions of stakeholders

**DOI:** 10.1371/journal.pone.0219686

**Published:** 2019-07-18

**Authors:** Thando Katangwe, Hannah Family, Jeremy Sokhi, Hiyam Al-Jabr, Charlotte L. Kirkdale, Michael J. Twigg

**Affiliations:** 1 School of Pharmacy, University of East Anglia, Norwich, United Kingdom; 2 Boots UK, Nottingham, United Kingdom; 3 Medical School, University of Bristol, Bristol, United Kingdom; University of Ghana College of Health Sciences, GHANA

## Abstract

**Background:**

Diabetes prevention programmes delay or prevent the onset of type 2 diabetes in people with pre-diabetes. To increase accessibility, national guidelines recommend delivering diabetes prevention programmes in primary care settings, including community pharmacy. This study aimed to explore the English community pharmacy setting as an option for delivering diabetes prevention services.

**Methods:**

Two focus groups and nine semi-structured interviews were conducted with stakeholders including, community pharmacists, general practitioners and commissioners. The topic guide was framed using the COM-B theoretical model for behaviour change to elicit practitioners’ capability, opportunity and motivation to engage with providing or referring to community pharmacy diabetes prevention services. Data were analysed thematically, and barriers/facilitators mapped to the COM-B framework.

**Results:**

Five themes were identified: ‘Pre-diabetes management and associated challenges’, ‘The community pharmacy setting’, ‘Awareness of community pharmacy services’, ‘Relationships and communication’ and ‘Delivery of community pharmacy services’. Community pharmacy was highlighted as an accessible setting for delivering screening and follow-on lifestyle interventions. Key factors for enhancing the capability of community pharmacy teams to deliver the interventions included training and appropriate use of skill mix. Delivering diabetes prevention services in collaboration with general practices was identified as key to the provision of integrated primary care services. Whilst financial incentives were identified as a motivating factor for delivery, service promotion to patients, public and healthcare professionals was perceived as crucial for enhancing engagement.

**Conclusions:**

This research highlights a role for community pharmacy in diabetes prevention. New service models should seek to integrate community pharmacy services in primary care to facilitate patient engagement and better communication with general practices.

## Introduction

In England, approximately five million people have pre-diabetes [1]. It is estimated that 5–10% of people with pre-diabetes develop type 2 diabetes every year, although this may vary with population characteristics and pre-diabetes definitions [2, 3]. Evidence indicate that early identification of individuals with pre-diabetes and subsequent implementation of behaviour change related to diet and physical activity can significantly reduce progression to type 2 diabetes [4]. However, systematic review evidence suggests that the impact of diabetes prevention programmes (DPPs) could be undermined by poor engagement amongst people with pre-diabetes [5].

A National Health Service DPP (NHS DPP) in England, which aims to identify people with pre-diabetes and refer them onto a behavioural change group-based intervention, was implemented in 2016 [6]. A recent update on the programme reported a post-referral attendance rate of 49% to the initial assessment [7]. Qualitative evidence exploring engagement with DPPs has identified possible barriers to include work and social commitments, inconvenient location and session times and transportation [8–10].

Primary care settings demonstrate the greatest reach to people with pre-diabetes [11]. In England, community pharmacy is the most visited NHS primary care setting, with approximately 90% of the population having access within a 20 minute walk [12]. Evidence investigating the implementation of DPPs in community pharmacy settings has demonstrated feasibility in the delivery of both screening and lifestyle-change interventions [13, 14]. In countries such as the USA, where a national DPP has been implemented for a number of years, clear guidelines outlining community pharmacy involvement in pre-diabetes screening and delivery of DPPs have been developed [15].

In England however, with pre-diabetes primarily identified through routine primary care appointments or retrospective screening of general practice databases, the role of community pharmacy in the delivery of the program remains undefined [16, 17]. Additionally, although community pharmacy delivers opportunistic screening and refers to mainly general practice services [18], there are currently no routine lifestyle interventions being delivered in this setting for people with pre-diabetes. Nor are there clear guidelines for how community pharmacists could deliver lifestyle interventions for this population. Therefore, with the NHS long term plan advocating involvement of community pharmacists in primary care networks for case finding and treating high risk conditions [19], it is important to establish a clear role for community pharmacy in the national programme and determine whether it could increase reach to this population. Additionally, there is a need to better understand the likely barriers and facilitators to delivering public health interventions in this setting from the perspective of multiple stakeholders including community pharmacy teams, general practice teams and commissioners.

Successful delivery of public health interventions such as DPPs in this community pharmacy would require behaviour change at many levels including individual (pharmacists), organisational (community pharmacy) and community (primary care and local communities) [20]. In this study we applied the COM-B, a theoretical model which recognises that behaviour change is brought about by interacting components including Capability, Opportunity and Motivation, to understand the key determinants for ‘the delivery of diabetes prevention services (DPS) by community pharmacy teams’ [21]. The aim of this research was therefore to explore the community pharmacy setting as an option for delivering DPS by eliciting views of stakeholders and using the COM-B model to frame the data collection, analysis and future direction of interventions aimed at patients and healthcare professionals.

## Methods

### Study design

This is a qualitative study that adopted a pragmatic epistemology and used semi-structured interviews and focus groups to explore the study aims with various stakeholder groups [22]. Ethical approval was obtained from the Health Research Authority (IRAS project ID: 233631) and the Faculty of Medicine and Health Sciences Research Ethics committee at the University of East Anglia before commencing the research. The study took place in Norfolk, UK between January and March 2018.

### Rationale for study design

A pragmatic and exploratory approach was used to address this research topic in which very little research has previously been undertaken [22, 23]. Pragmatism, a philosophy that recognizes that there are different ways of interpreting the world and research, suggests there to be multiple realities and hence that no single point of view can ever give the entire picture [24, 25]. Pragmatic research therefore seeks to use whatever combination of methods necessary to find the answers to research questions. This study adopted the use of both focus groups and interviews to explore the research topic with multiple stakeholders. Focus groups were deemed central to exploring the research topic in the selected group of participants who often work as a team to deliver services [26]. However, in order to provide flexibility to potential participants and thus encourage participation, the interview option was made available to GPs, nurses and commissioners. This option was also used to support an honest in-depth account of experiences and opinions about community pharmacy and community pharmacy teams from this group of participants.

### Study setting

This study was set in primary care, specifically community pharmacy and general practice settings [27]. General practices are private healthcare businesses that have an important role in providing healthcare to local communities. In the UK, although the majority of general practices work to NHS contracts, follow NHS guidelines and see NHS patients, they do not compete for patients, or profit in the way privately funded providers of healthcare do. General practices consist of multidisciplinary teams including general practitioners (doctors), nurses and pharmacists and are responsible for both looking after patients with chronic illness and health promotion. Community pharmacies are also private healthcare providers who work to NHS contracts to provide medicine related services such as dispensing and counselling. As part of their contract community pharmacies also provide health promotion services such as weight loss and smoking cessation programmes.

In England, local health promotional services provided by both general practices and community pharmacies are commissioned by Clinical Commissioning Groups (CCGs)[28]. Clinical commissioning groups are groups of general practices which come together in an area to commission the most appropriate services for their patients and population. These groups therefore buy services for their local community from any service provider, including community pharmacy, which meet NHS standards and costs. Commissioners are usually supported by Clinical Support Units with external support, specialist skills and knowledge and may also consult Local Pharmaceutical Committees (LPCs), who represent all pharmacy contractors in a defined area, on services that could potentially be provided via community pharmacy.

This study involved multiple stakeholders involved in both the provision and commissioning of local health promotional and preventative services in order to obtain a more complete perspective on a potential role of community pharmacy in delivering DPPs in primary care.

### Participants

Eligible participants were community pharmacy personnel, general practitioners and nurses working in the UK. Community pharmacy personnel included pharmacists and technicians involved in the delivery of public health services. General practitioners, nurses and other pharmacists were only eligible if they were working for general practices participating in pre-diabetes screening and referral to the NHS DPP and had a special interest in diabetes. Individuals involved in commissioning and negotiating services for community pharmacy were also eligible to participate in the study.

### Participant identification and approach

Research information was circulated to potential participants in community pharmacies and general practices via emails sent through area, store and practice managers. Commissioners were identified and sent research information through the Research and Development office and/or existing contacts.

Participants involved in focus groups and interviews conducted outside of working hours were reimbursed for travel costs and received a £30 voucher for participating. General practices were reimbursed at £80 per hour for GP time and £23.21 per hour for nurses’ time for interviews conducted during working hours. Participating commissioners declined the offer of a voucher at £30 per hour, instead choosing to participate for free.

### Sampling

The study aimed to conduct two focus groups and a maximum of 10 interviews. To ensure a good representation from chain and independent pharmacies recruiting of community pharmacy participants involved purposive sampling based on job titles and workplace [26]. We aimed to achieve a focus group size ranging from 5 to 8 participants [26]. Convenience sampling was used to recruit commissioners, GPs and nurses. All GPs, nurses and commissioners opted for interviews rather than focus groups, hence focus groups were only conducted with community pharmacy participants.

### Data collection

Semi-structured interviews were conducted at the University of East Anglia or participants’ workplace by the main researcher (TK) and lasted up to a maximum of 30 minutes. Focus groups were held at the University of East Anglia and facilitated by the main researcher (TK) and another member of the research team and lasted approximately 60 minutes. Both interviews and focus groups were digitally audio recorded. Written consent was obtained from all participants.

### Topic guide

The semi structured topic guide used to facilitate data collection for both interviews and focus groups is summarised in Table 1. It was developed based on a review of literature, discussion among the research team and underpinned by the COM-B theoretical model [21]. The topic guide was tailored to the appropriate healthcare professional group or commissioner, but the key issues remained the same.

**Table 1:** Topic guide summary.

Research topic	Issues discussed
**Background**	• Current job role and work experience
**Pre-diabetes (where applicable)**	• Experience with the management of pre-diabetes
**Community pharmacy services**	• Experience and views about current community pharmacy services • Views on current primary care based public health services e.g. NHS Health Checks
**Community pharmacy-based diabetes prevention**	• Views on the role of community pharmacy in diabetes prevention • Capability: barriers and facilitators for using **community pharmacy personnel** to deliver diabetes prevention services • Opportunity: barriers and facilitators for using the **community pharmacy setting** for delivering of diabetes prevention services • Motivation: barriers and facilitators for community pharmacy teams **delivering diabetes prevention services as part of the primary care team**

### Analysis

Interviews and focus group recordings were transcribed verbatim by the main researcher (TK) or a paid contractor. To provide an iterative process of analysis Braun and Clarke’s six phases of thematic analysis were conducted [29]. The transcribed data were re-read and inductively coded by the main researcher (TK). Relationships between the codes were sought to develop subthemes and subsequent themes by two members of the research team (TK and HA). Codes and themes were checked by another member of the research team (MT) and any disagreements resolved by consensus, referring to the transcripts.

To facilitate a theory informed analysis, themes associated with the target behaviour (i.e. the community pharmacy team delivering DPS) were identified by two members of the research team (TK and MT). Respective codes from the themes were then separated into barriers and facilitators and mapped onto the three domains of the COM-B model i.e. capability, opportunity and motivation. Mapping was carried out independently by three researchers (TK, HA and MT). Following this, the mapping was further checked by another member of the research team (HF) with a psychology background and experience in using the COM-B. Any disagreements were resolved by consensus, referring to the codes and original transcripts.

## Results

Two focus groups (N = 7 and N = 5) with community pharmacy participants and 9 interviews with GPs, nurses and commissioners were conducted. Participant characteristics are summarised in Table 2. Thematic analysis identified the following five main themes: ‘Pre-diabetes management and associated challenges’, ‘The community pharmacy setting’, ‘Awareness of community pharmacy services’, ‘Relationships and communication’ and ‘Delivery of community pharmacy services’. The first theme sets the context for the current management of people with pre-diabetes in primary care which is largely carried out in general practice whilst subsequent themes relay factors associated with delivering DPS in community pharmacy. What follows aims to provide a narrative on the first theme to provide context, followed by the COM-B analysis of the subsequent themes.

**Table 2:** Participant characteristics.

Characteristic	Total (N = 21) N (%)
**Gender**	
• Female	16 (76.2)
**Profession**	
• Pharmacist (registered)	8 (38.1)
• Pharmacist (pre-registration)	1 (4.8)
• Pharmacy technician	3 (14.3)
• General practitioner	3 (14.3)
• General practice pharmacist	1 (4.8)
• Nurse	3 (14.3)
• Commissioner (pharmacist)	1 (4.8)
• Commissioner (non-healthcare professional)	1 (4.8)
**Place of work**	
• Pharmacy chain	9 (42.9)
• Independent pharmacy	3 (14.3)
• General practice	7 (33.3)
• Commissioner (Local Pharmaceutical Committee-non-healthcare professional)	1 (4.8)
• Commissioner (Commissioning Support Unit—pharmacist)	1 (4.8)

### Theme 1: Pre-diabetes management and associated challenges

General practice participants largely welcomed the NHS DPP as a referral option that saved them time and allowed them to focus on other conditions. These participants reported positive feedback from patients who had engaged with the programme with respect to weight loss and lowering HbA1c. However, despite the implementation of the NHS DPP, there was a variation in its utilisation by participants working in general practices who described using different pre-diabetes management protocols. GP and nurse participants described providing diet and lifestyle advice using, but not limited to, leaflets and face to face or telephone consultations.

*“It is a good option* [NHS DPP] *I do feel because of the time element and obviously we’re really busy in primary care*. *Whilst I would always offer that time to the patient equally if they say*, *‘yes I will go on the diabetes prevention’*, *that does then reduce that*, *not burden*, *but it transfers that responsibility over”* **[P18-Nurse]**

Experience with referral to the NHS DPP was also varied amongst GP and nurse participants. Whilst most GP participants felt that people with pre-diabetes were generally receptive to their referral to the NHS DPP, most nurse participants felt that uptake was low and largely affected by location and transportation. Apart from accessibility, other barriers to participation included social and work commitments, a dislike of group-based sessions and patients’ perceptions that they had adequate knowledge and capability to make changes themselves. Some nurse participants also felt that engagement was noticeably low amongst people with co-morbidities and those from low socioeconomic backgrounds.

*“The other thing is a lot don’t like groups…the minute I found that I say oh you know it’s a group session*, *they say*, *‘oh no I don’t want to go*, *I don’t do groups’”* **[P16-Nurse]**

### COM-B analysis

Four themes, briefly described below, were directly related to the target behaviour ‘community pharmacy teams delivering DPS’ and thus included in the COM-B analysis. The separation of the codes in each theme into barriers and facilitators, illustrative quotes and mapping onto the Capability, Opportunity and Motivation domains is presented in Table 3 together with the descriptions of the domains.

**Table 3:** COM-B analysis of barriers and facilitators to delivering community pharmacy-based diabetes prevention services.

COM-B components with definitions	Mapped codes		Illustrative quotes
	Barriers	Facilitators	
**Physical capability** (Physical skill, strength or stamina)		• Practical training	*“I think if the CCG is commissioning a service then they should be able to provide us with the practical training”* **[Pharmacist]**
**Psychological capability** (Knowledge or psychological skills, strength or stamina to engage in the necessary mental processes)	• Inadequate training to deliver services	• Knowledge of support staff • Consultation skills • Coaching and behaviour change skills	*“I think we need to be very mindful that when we’re training our staff it’s not just about how you use the equipment*. *We have to up-skill them on consultation skills as well*, *because if people are to be utilising us more*, *they also need to feel that they’re getting quality service”* **[Pharmacist]**
	• Maintenance of knowledge/skills is important		*“You need the skills to be concentrated because if like say for example in the past we* [GP practice] *used to provide smoking cessation services*, *but we felt that we were not dealing with enough number of services so that our skills would remain at a high level”* **[GP]**
**Physical opportunity**–(Opportunity afforded by the environment involving time, resources, locations, cues, physical affordance)		• Accessibility	*“It’s about access as well*. *I think access is very important because I’ve had customers*, *they would have gone to the GP otherwise if we weren’t closer… one of them had to go in a wheelchair on the bus to go all the way to the surgery whereas they could just leave the house go in the wheelchair to the pharmacy and have it* [Flu vaccination] *done and then go home*, *so for them it’s easy access”* **[Pharmacist]**
		• CP setting well placed to deliver pre-diabetes services	*“How easy would it be to actually do things like mass screening in community pharmacy and the answer is really really easy…community pharmacy could be picking up pre-diabetics and you know giving the intensive lifestyle advice*, *weight management etc*. *you know that’s such a piece of cake*” **[Commissioner]**
		• CP screening for NHS DPP could deliver faster referrals than surgeries	*“I think it could only be a good thing for everybody because the delay in patients getting appointments in a busy practice means that if they are able to go via the pharmacist then they would get the referral quicker than perhaps waiting for an appointment to see somebody here to then be referred into the system”* **[Nurse]**
		• Appointment systems with shorter waiting times than general practice • Walk in services	*“Actually*, *booking appointments*, *I think*, *works for a lot of people even if they have to wait ten minutes*. *I think that’s better than what they have to wait at the doctors surgery’s”* **[Pharmacy technician]**
		• A time-flexible alternative	*“I think it’s again going back to individualisation…some patients would chose not to engage in the prevention programme*, *they may feel I don’t want to go to my GP surgery*, *I can’t ever get an appointment or I don’t have time to go there because their lifestyle and choices and things*. *So if they are willing to engage with their local pharmacy I would say its surely better that they engage with somebody and receive that advice and education that they need than getting signposted to somewhere that they are not going to follow-up with and not get any education at all”* **[Nurse]**
	• Time pressure barrier to delivering diabetes prevention services • Pharmacist time constraints hindering delivery of services		*“I can see this eruption this volcano erupting and suddenly not only will general practice be overwhelmed but so will the pharmacist delivering one to one because its very time consuming”* **[Nurse]**
	• Time pressures leading to low quality service delivery	• Delivery of public health services need adequate time	*“With diabetes our main problem is that we don’t have time of such for these kind of things we do them of course but there are a lot of time restraints that limit of us to the sort of quality that we may be able to give our patients with the services”* **[Pre-registration pharmacist]**
	• Space challenges		*“In terms of other barriers some pharmacies it would be their consultation rooms aren’t necessarily ideal”* **[Commissioner]**
	• Lack of access to medical records		*“The only thing I would say is that I don’t see how a pharmacy can help with medication reviews and tell patients they shouldn’t be taking certain drugs when they don’t have access to their blood results for some cases [laughter]”* **[Nurse]**
	• Funding cuts a barrier to CP delivering more services	• Future CP services would need to be well funded	*“You know what 6% shaved off*! *I mean that 6 seems like a small number but that’s big money you know because it’s paying for your staff to be able to deliver these services so that’s what it comes down to…we’re in this difficult situation right now… we want to be doing more we want to be involved more and like we’re tied*, *really we’re tied to the dispensary*, *we’re tied to these prescriptions”* **[Pharmacist]**
	• Lack of resources to deliver beneficial services		*“To give those services out and be beneficial to the patients a second pharmacist is always good* .* *.* *.*I mean we’ve got a second pharmacist in in our pharmacy for at least 4 days a week haven’t we but they said you know they are trying to that is getting harder and harder to fund”* **[Pharmacy technician]**
	• Current CP services not Integrated in primary care • Pharmacists cannot deliver DPS without general practice • Perceives CP diabetes prevention services as fragmentation of primary care services	• Integration in primary care • Commissioning model and integration fundamental • CP and GP need to work together more • General practice should refer patients into new CP services	*“The issue with all community pharmacy services at the moment is that they are not integrated at the end of the day they are an afterthought a bolt on…work separately”* **[Commissioner]**
	• Current follow-up systems not efficient • Lack of feedback from CP services hindering referrals • Poor feedback from GP practice following CP referrals • IT systems not merged with GPs hindering GP referrals, follow-up and leading to duplication of work	Effective communication, feedback and referral systems to general practice are needed for the delivery of services IT connectivity fundamental for CP-GP integrated services	*“You need the IT solutions etc*. *to be able to pass that information back to the GP practice*, *because at the moment it’s not an integrated system*. *So IT connectivity and read write abilities etc*. *are kind of fundamental I think to the integration of community pharmacy service going forward”* **[Commissioner]**
**Social opportunity** (Opportunity afforded by interpersonal influences, social cues and cultural norms that influence the way that we think about things e.g. the words and concepts that make up our language)	• Challenges in funding services traditionally provided by general practice • No dedicated budget pot for commissioning CP services		*“One of the problems at the moment with the way that commissioning happens in the NHS in primary care is if we are commissioned to do something that is a job that traditionally might have been done by the GP practice*, *how do you release that money*?. *You are not going to de-commission the GP practices*, *you’re not going to take money away from them etc*. *so how do you then fund that work that is being transferred to community pharmacy*?*”* **[Commissioner]**
	• Commissioners do not prioritise CP • Pharmacy underrepresented in CCGs • Commissioners envision primary care as primary medical care (which doesn't include CP)		*“I think the biggest barrier to developing community pharmacy services is the fact that commissioners at a local level do not see it as priority”* **[Commissioner]**
		• Increased awareness • Targeted awareness • CP services awareness—responsibility of all HCP including CP	*“I think the diabetes prevention program would be another good service we provide though provided we create the awareness so that people would know we are doing that*, *we’ve got the training to do that”* **[Pharmacist]**
	• Patient barriers—only wanting to engage with prescription services	• Need positive promotion of CP i.e. not as cheaper alternative but accessing right level of care • Patient need to move in with the times and start using other HCP more rather than expecting to see GP	*“I think also the raising of awareness of pharmacy need to be in a positive way*, *because you know the stuff that I’ve seen around pharmacy has been you know doctors too busy so go and see your pharmacist*, *or medicines are costing too much money go buy them cheaper in the pharmacy*, *and so I’m not 100% sure that that message is wholly positive”* **[Pharmacist]**
	• Ethical challenges with promoting CP services		*“Then again there’s another point with private companies like [pharmacy multiples] trying to advertise for services*. *It’s like this is a health thing do I really advertise it like I’m advertising for maybe perfume or milk*? *There’s that ethical aspect”* **[Pharmacist]**
	• Lack of awareness of CP services (GP) • GP only aware of pharmacist role in medication • Lack of knowledge of CP role and skills		*“I think that GP’s don’t understand*, *have no idea what pharmacists know and what pharmacists could do in community pharmacy… it’s just a lack of knowledge about that”* **[GP practice pharmacist]**
	• Sceptical if prevention service is feasible in CP setting Sceptical if CP is the best setting for delivery of diabetes prevention advice		*“I mean if they’ve got the appropriate resources then I can’t see any major disadvantages*, *but whether it’s feasible to provide all these services in a pharmacy setting I am not so sure*, *and whether one person can do all these things am not so sure”* **[GP]**
	• Sceptical about follow-up following screening in CP • CP public health screening services with no follow-on programmes wasting primary care resources		*“In terms of screening I can’t see any reason why it can’t be done outside of the surgery setting but I am a bit sceptical about how that would be dealt with in by the pharmacist*. *Meaning is it going to be a case of them just doing a blood test and then if they’ve got an HbA1c of 42 say oh go and see your GP or whether they can then give any focused advice about that or whether they would be empowered to do the necessary referrals to the say for example the diabetes prevention programme”* **[GP]**
	• Commissioning CP services difficult due to multiple contractors	• Commissioning for outcomes better model of demonstrating impact of service	*“They need to know what we they are commissioning and commissioning for outcomes… unless you can say what you are going to deliver and performance manage it then you know it’s always going to be questionable as to the impact that you’re providing”* **[Commissioner]**
	• Commissioning CP services difficult due to multiple contractors		*“Obviously we’ve got yes some big providers like [name of pharmacy multiples]… but we’ve also got individuals and if you were an evolving care organisation…an accountable care organisation and you wanted to commission something like that from community pharmacy…*.*how do you manage it…in an area might be 30*, *40*, *50*, *60 different contractors… so you need a vehicle really to actually deliver that”* **[Commissioner]**
	• Competing interest in delivering services Competing interest with GP practices for services		*“With regards to services moving out of primary care*, *if GPs provide the screening services then we get* .* *.* *.*as I said to you earlier we get kind of paid for it and it’s a source of income*. *So even though it might not be a huge source of income but because of the precarious state a lot of GP are around the country even smaller reduction in their income will have a destabilising effect”* **[GP]**
	• Competing interest affecting CP-GP relationships		*“There is some competition between services especially the flu vaccination… there’s been quite a lot of inappropriate advertising from both sides in the past few years to try to get patients so that’s something that kind of ruins the relationship a little bit”* **[Pharmacy technician]**
	• GP perceiving that CP has an ulterior motive for providing services • Perceives CP delivering pre-diabetes advice as stepping on GPs toes	• DPP would need to be positively promoted to practices to ensure they don't see it as challenge upon their services	*“Our satisfaction rates are have always been high in spite of whatever the newspaper say… and that's because we feel that the patients feel that we are doing what we are doing for them rather than for any other ulterior motive*. *I guess when they going to see a pharmacist even if they are very altruistic*, *even if they want to be just doing good for the patients*, *there always the suspicion if is it really just for me or is it because they are after their bottom line yeah so I don’t know”* **[GP]**
		• Pre-diabetes education not efficient use of GP time	*“We were referring patients to the health trainer…anyone who was diagnosed with [pre-] diabetes was sent her way because it’s not actually it’s not efficient use of our time to really educate somebody with pre-diabetes”* **[GP]**
	• GP practices not referring patients to CP public health services		*“There is an awful lot of surgeries that can't engage because they are busy as well and can't and don’t want to engage but they are not necessarily referring patients to community pharmacy”* **[Commissioner]**
	• Potential patient resistance because historically they would see a nurse or a GP for diabetes services	• GP endorsement of CP services would positively influence uptake • GP endorsement of CP DPP would be important for instilling confidence in patients	*“If the GP’s were to promote pharmacy then I think a lot more people will be more willing to uptake services”* **[Pharmacist]**
		• CP could help reduce GP workload	*“I think that’s good because from our point of view as primary care and GP practice were trying to reduce our footfall as much as possible in terms of patients coming into the surgery for things that can be dealt with by pharmacies”* **[Nurse]**
	• CP time pressure leading to unwarranted referrals to general practice • CP public health screening services creating more referrals and workload for general practice		*“If they are doing those things we need to see it…referring back if we need to something the only problem with that is that its more workload for us but it’s only the same as someone getting a private medical and then we have to deal with that so”* **[GP]**
	• Fear of overwhelming working environment that CP DPS could create in primary care		*“I can see this eruption this volcano erupting and suddenly not only will general practice be overwhelmed*, *but so will the pharmacist delivering one to one”* **[Nurse]**
	• Poor relationships with pharmacy multiples	• Positive working relationships with general practice-owned pharmacies • Good referral systems depending on relationships	*“I suppose because we have got our own pharmacy we just work through …yes so we know them all so they are employed by the practice so we’ve got pharmacy patients and dispensary patients so it’s all done within the practice”* **[GP]**
		• GPs need to have confidence in pharmacy team ability to deliver DPP	*“It’s you know trying to build the confidence of the doctors in us as well and our teams because at the end of the day if we do something like this it’s unlikely it’s going to be use that’s delivering the service it’s going to be our healthcare team so they have to build up confidence in what we’re doing”* **[Pharmacist]**
		CP need to build trust with GPs	*“Yeah I mean I guess there ought to be a bit more kind of trust in between*, *I think it’s mostly a trust issue*. *If GPs are to trust that what they are doing they are doing it properly and then the GPs don’t have to take up the extra burden but not be paid for it*, *then I think it would work well”* **[GP]**
	• Potential resistance from general practice because historically patients go to a GP setting for diabetes services		*“I would imagine that there could potentially be some resistance from obviously places like us as a GP setting*, *because historically it would always be that you came to your GP and you know if the GP or the practice nurse or whoever would see you and diagnose you and give you advice and so on”* **[Nurse]**
	• GPs perceiving to be better than pharmacists at giving pre-diabetes due to extensive knowledge of diabetes and associated co-morbidities • GPs perceiving to be better placed to give pre-diabetes opportunistic advice due to links with co-morbidities in patients the consult		*“I think the background knowledge is very important but what is also important is the experience behind it*. *I mean it will be very difficult for a pharmacist to replicate the experience which a GP will have because diabetes is not just diabetes*, *its kidney disease*, *its heart disease*, *its peripheral vascular disease and we see it day in and day out*. *I think a pharmacist will be adjunct to this but I don't think pharmacists will be able to do this all on their own*.*”* **[GP]**
**Reflective motivation** (Reflective processes involving plans (self-conscious intentions) and evaluations (beliefs about what is good and bad))		Use pharmacy skill mix to deliver diabetes prevention services CP public health interventions don’t have to delivered by pharmacists	*“We are supposed to be utilising and making best use of the skills mix … because as much as we get frustrated with the monotony of our role as do our dispensers and our healthcare assistants so introducing these things can make them feel challenged and provide opportunities for growth”* **[Pharmacist]**
	• Dispensary role of pharmacist hindering scope to deliver more services • Pharmacy workload hindering delivery of services	Appropriate allocation of resources	*“Our employers have to be on-board properly*. *We need the support unless this can be done by a designated member of staff*, *but if it’s on the pharmacists again then that would be a problem because as it is there is so much that I need to do”* **[Pharmacist]**
	• Inadequate training leading to lack of confidence	Self-efficacy of staff in delivering services enhanced by training and experience Confidence of patient and GPs on CP delivering services enhanced by training and experience	*“I think it’s imperative that you know the services are standardised across the board that will instil confidence ok for us and also for the patients you know you don’t want your patient to come in and you don’t know what you’re doing”* **[Pharmacist]**
	• Lack of structure to deliver particular services leading to pressure on pharmacist resources • Overwhelming experience created by unstructured delivery of CP services		*“If you get people come marching through your door to speak to your pharmacist*, *and as you were saying you’ve got your methadone addicts*, *and you’ve got your morning after*, *and you’ve got your MUR’s*, *it sometimes as a pharmacist you don’t know where your backside is really because you're everywhere”* **[Pharmacist]**
		Implementation of service with GP to alleviate tensions caused by competing interests	*“The worry is if the GP’s think oh you’re just taking their job away…so it’s trying to make sure that we get a good conversation going with the GP’s and actually come up with a good way to actually implement the service with them”* **[Pharmacist]**
		Delivering pre-diabetes lifestyle advice does not require one to have a medical degree	*“As a GP I mean I do do an awful lot of it [lifestyle advice] opportunistically within the consultation because it relates to so many things… blood pressure and anything but you don’t need a medical degree to give lifestyle advice”* **[GP]**
**Automatic motivation** (Automatic processes involving emotional reactions, desired (wants and needs), impulses, inhibitions, drive states and reflex responses)	• GPs will only endorse services if there something in it for them		*“If obviously the doctors have got QOF targets and they will be paid for a similar thing then they’re not going to be sending people to me if they can get that money isn’t it”* **[Pharmacist]**
		CP diabetes prevention services would bring in financial benefits	*“So cost wise in providing the service I think it would be cheaper for the NHS for us to do it [deliver DPS] than to get the GP surgery’s to do that…also hopefully they will channel a little bit of money you know from there into the community pharmacy so that they can provide us with extra hands that we need”* **[Pharmacist]**
	• Pharmacists intimidated by GPs—affecting relationships		*“I think as pharmacists we can find it you know really difficult to talk to GP’s sometimes… I think of what I used to be like with consultants*, *they seemed you know they were up here…that’s a personality thing sometimes and I think it would be the same”* **[GP practice pharmacist]**

#### Theme 2: The community pharmacy setting

This theme largely discussed physical characteristics of the setting such as accessibility in relation to engagement of people with pre-diabetes with DPS. Barriers and facilitators related to delivering DPS in community pharmacy included time and resources and as such were mapped to the physical opportunity domain.

#### Theme 3: Awareness of community pharmacy services

This theme considered the societal role of community pharmacy in public health and primary care. The theme, largely discussing the level of awareness of community pharmacy services by the public, patients and other healthcare professionals, identified barriers and facilitators which were primarily mapped to the social opportunity domain.

#### Theme 4: Relationships and communication

This theme discussed communication challenges between community pharmacies and general practices and the impact of relationships in enhancing and hindering communication and delivery of services. Barriers and facilitators relating to this theme were mapped onto the opportunity and motivation domains.

#### Theme 5: Delivery of community pharmacy services

This theme explored the practical aspects of delivering public health services, including DPS, in community pharmacy. The theme considered the capability of community pharmacy teams, the availability of physical resources and the motivation behind wanting to engage with delivering the services. Hence the theme contributed to all three domains.

### Capability

Training was identified as the main enabler for enhancing capability of community pharmacy teams to deliver DPS. Whilst most participants perceived pharmacists to have adequate knowledge to deliver DPS, they felt other team members, such as technicians and dispensers who work under supervision of pharmacists, would need a sound theoretical understanding of pre-diabetes and its management. Participants felt that this was crucial for giving other team members autonomy, subsequently requiring less pharmacist intervention. Practical training was also perceived to be crucial for all members of the team including pharmacists.

*“I think if the CCG is commissioning a service then they should be able to provide us with the practical training”* **[P4-Pharmacist]**

Other training requirements highlighted as important for supporting people with pre-diabetes in the making desired lifestyle changes included coaching, behaviour change and consultation skills. In general, most participants felt that, with training, any personnel including community pharmacy teams could deliver DPS.

*“I’m sure we’ve had consultations whether it be with a healthcare assistant or a nurse or a doctor where we think*, *‘that could have been a little bit better’*, *and so I would want to ensure that when people are coming into our pharmacy that they’re having a positive experience with the member of staff who is delivering the services to them”* **[P8-Pharmacist]**

### Physical opportunity

Community pharmacy was perceived as well-placed for delivering pre-diabetes screening services that could afford a faster referral pathway into the NHS DPP. Accessibility was considered as an enabler for engagement of people with pre-diabetes, with key setting characteristics including location and the provision of walk-in services.

*“Well for a start we are more accessible*. *We open seven days a week…it’s not like Monday to Friday the GP’s…they [patients] can come in over the weekend and see someone as well*. *It might be a good thing [to deliver DPS]”* **[P5-Pharmacist]**

In considering the practical delivery of DPS, community pharmacy participants identified time as a key facilitator. Participants felt that delivering public health interventions requires adequate time and resources, which when compromised, often lead to low quality, “tick box” services. The lack of access to full patient medical records and IT systems which are not merged were considered as barriers to efficient communication and referrals between community pharmacy and general practice.

“You need the IT solutions etc. to be able to pass that information back to the GP practice because at the moment it’s not an integrated system. So IT connectivity and read write abilities etc. are kind of fundamental I think to the integration of community pharmacy service going forward”

### [P20-Commissioner]

A major concern highlighted by community pharmacy participants and commissioners was the current funding cuts and the lack of dedicated budgets for services commissioned in this setting. It was therefore felt that reasonable reimbursement would be required to account for the time and resources invested in delivering future services.

*“The problem is the chicken and egg*. *Does pharmacy develop and staff itself for those services*, *but how does it do so before the funding and everything becomes available*?*”* **[P20-Commissioner]**

### Social opportunity

Community pharmacy was considered to have potential for increasing patient centred care by providing more choice. Participants felt community pharmacy could increase reach to men and regular pharmacy users due to the settings’ propensity for normalising care and the non-judgemental and anonymous environment it provides. It was also seen as suitable for accommodating an individualised intervention as an alternative to the current group intervention offered in the national DPP.

*“I think another benefit [of community pharmacy-based DPS] is also that they develop that link with their pharmacist*. *I guess perhaps that would be it*, *that if you’ve got somebody that’s on quite a few medications anyway they’re used to going to the pharmacist*, *it’s not a big deal”* **[P19- GP practice pharmacist]**

Although community pharmacy participants considered the delivery of DPS to be part of their public health role, they felt there is a general lack of awareness of this role amongst patients, the public and other primary care teams. This resonated amongst general practice participants who, although aware of medicine-related services, seemed unaware of the range of public health interventions delivered in community pharmacies. Additionally, commissioners and some community pharmacy participants expressed concerns that NHS promotional campaigns had so far presented community pharmacy as a cheaper alternative to general practice. These participants were referring to ‘Stay Well Pharmacy Campaign’ launched in 2018 to encourage the public to visit their local pharmacy team first for clinical advice for minor health concerns [30]. This campaign was launched in a climate in which millions of GP appointments and visits to emergency services were for treatable conditions and estimated to cost the NHS more than £850m each year [31]. Therefore, although the key message of the campaign was that community pharmacists and technicians are qualified healthcare professionals and well suited to meet the clinical need, these participants perceived the underlying message of the campaign, which is that using pharmacy for minor concerns will free up GP time for more urgent appointments and save NHS money, to be more prominent. These participants conveyed the need for promotion centred on accessing the right level of care.

*“If you change the message to*, *‘you’re still going to get primary care services you’re just accessing it at a more appropriate place’*, *it’s a different message and it might drive behaviours to change because as a patient if you get told you are going to see the cheap alternative you might not want to go there”* **[P21-Commissioner].**

The delivery of DPS such as screening and lifestyle programmes as part of the primary care team was also discussed. Community pharmacy participants felt that service endorsement by GPs and nurses involved in the diagnoses pre-diabetes was crucial to service uptake. However, some participants felt that endorsement of, and referral to, community pharmacy services by general practices was largely dependent on working relationships.

Some participants felt that the delivery of DPS in community pharmacy could generate resistance from both GPs and patients. To this end some participants described how screening services which mainly refer to general practice for confirmatory tests, could create extra workload and negatively affect their revenue. One GP in particular felt disadvantaged by current screening services which refer patients at high risk of cardiovascular diseases or diabetes to them as they felt that community pharmacy was getting paid to do the easy part whilst general practices were left to deal with the long-term management of the conditions for no extra payment. For this reason, the participant expressed a need for pharmacists to be empowered to do thorough screening tests requiring no referral for confirmatory tests and that community pharmacy teams should also be empowered to either refer straight into the NHS DPP or provide follow-on preventative services. Although this view was not expressed by all, community pharmacy participants also acknowledged the lack of follow-on services in this setting.

*“If GPs are to trust that what they [community pharmacy teams] are doing*, *they are doing it properly and then the GPs don’t have to take up the extra burden but not be paid for it*, *then I think it would work well…with regards to services moving out of primary care*, *I mean*, *if GPs provide the screening services we get kind of paid for it and it’s a source of income*. *So even though it might not be a huge source of income but because of the precarious state a lot of GPs are around the country even smaller reduction in their income will have a destabilising effect”* **[P14-GP]**

### Motivation

Motivation enablers for delivering DPS as part of the primary care network included incentives. Community pharmacy participants also felt that, to avoid competition, future services should offer benefits for general practices as an incentive for them to endorse community pharmacy services.

*“It will depend on*, *if obviously the doctors have got QOF targets and they will be paid for a similar thing then they’re not going to be sending people to me if they can get that money isn’t it”* [Quality and Outcomes Framework—a reward and incentive programme for all GP surgeries in England, detailing practice achievement results] **[P5-Pharmacist]**

Self-efficacy, enhanced by training and experience, was also seen as fundamental for motivating community pharmacy teams to deliver DPS. Some participants felt that it was also important for other members of the primary care team, particularly GPs and nurses, to have confidence in community pharmacy’s ability to deliver the services. Participants also felt that self-efficacy would also increase patients’ confidence in community pharmacy’s ability to deliver DPS.

*“It’s you know trying to build the confidence of the doctors in us as well and our teams because at the end of the day if we do something like this it’s unlikely it’s going to be use that’s delivering the service it’s going to be our healthcare team so they have to build up confidence in what we’re doing”* **[P2-Pharmacist]**

The greatest barrier to motivation stemmed from pharmacists feeling overwhelmed in their current role. Participants felt that their dispensary role and the provision of largely walk-in services, could be a barrier to delivering DPS which are likely to require lengthy consultations. To this end participants felt that extra resources and improved utilisation of current skill mix, particularly technicians, would be required to deliver the services.

*“If you get people come marching through your door to speak to your pharmacist*, *and as you were saying you’ve got your methadone addicts*, *and you’ve got your morning after*, *and you’ve got your MUR’s* [Medicines Use Reviews], *it sometimes as a pharmacist you don’t know where your backside is really because you're everywhere”* **[P6- Pharmacist]**

## Discussion

This study highlights the potential for community pharmacy to deliver diabetes prevention services and presents factors in terms of Capability, Opportunity and Motivation at both local and national levels that could facilitate implementation.

The accessibility of community pharmacy has been identified in this study as a factor that could increase opportunity for people with pre-diabetes to engage with screening, glycosylated haemoglobin (HbA_1c_) monitoring and lifestyle interventions. A recent evaluation of the NHS DPP has recommended the programme be linked with other services in primary care and has highlighted the importance of increasing accessibility to targeted populations [32]. Therefore, with previous research demonstrating willingness amongst people with pre-diabetes to engage with DPS in community pharmacy (Katangwe T, 2019, unpublished data) and that people with pre-diabetes are more likely to be prescribed lipid lowering and anti-hypertensive drugs [33], community pharmacy could potentially have sufficient information to conduct focused screening and intervention services. However, since the lack of access to full medical notes was considered a barrier of delivering DPS in community pharmacy, the extent to which full access to medical notes would be needed to deliver the DPS would need to be established.

This study has also highlighted several important physical and social factors including time, resources and funding, that if addressed could enhance opportunity for community pharmacy teams to deliver DPS. This resonates with recent UK research which has demonstrated that despite the willingness of community pharmacy teams to deliver public health interventions, factors such as lack of time and funding remain major hindrances [34].

The need for integration of future community pharmacy services with other primary care services has also been identified. Factors affecting current integration in primary care such as the lack of integrated IT systems, poor relationships with general practices, competing payment structures and lack of awareness of community pharmacy roles and skills have been identified and would need to be addressed for future provision of community pharmacy services.

An independent review of community pharmacy clinical services commissioned by NHS England in 2015 also identified integration of community pharmacy within primary care as crucial for the provision of future services [35]. Potential solutions highlighted by both this research and the review include practical enablement such as shared clinical records and the ability to communicate with the rest of the clinical team [35].

An important finding of this present study, however, is a suggestion that current community pharmacy screening interventions such as NHS Health Checks [18], which refer high risk individuals to general practice services for further testing, could potentially be increasing general practice workload. A recent report on understanding general practice pressures has highlighted the changing relationship between general practices and the wider healthcare system as a contributor to workload and has highlighted referrals and communication as time consuming factors both for medical and administrative general practice staff [36]. It is important therefore that future community pharmacy services should seek to reduce pressure on general practice rather than increase it. Additionally, an evaluation of the NHS health check service has shown poor attendance amongst people referred to general practice services following screening in community pharmacy [18]. The evaluation demonstrated that almost half the people referred to other lifestyle interventions following community pharmacy services were unwilling to engage. This highlights that whilst some individuals are willing to engage with community pharmacy services, not all may be willing to engage with other primary care services.

Previous research conducted in Australian community pharmacies shows that risk assessments followed by fasting plasma glucose tests resulted in fewer referrals and greater uptake by patients [37]. More recent research conducted in Norwegian community pharmacies has further demonstrated the feasibility for community pharmacy to implement HbA_1c_ screening services [14]. With current guidelines for the diagnosis and referral into NHS DPP requiring HbA_1c_ screening,[17] there is potential for community pharmacy in England to be involved in delivering comprehensive tests without requirement for referral to other primary care teams for confirmatory tests. Moreover, with research also demonstrating potential cost-effectiveness of pre-diabetes screening with appropriate intervention in community pharmacy [38], lifestyle interventions for those unwilling to engage with other primary care lifestyle interventions could be delivered in this setting.

This study has highlighted training and the appropriate use of pharmacy skill mix as key factors that could enhance the capability and motivation respectively for the community pharmacy teams to deliver quality DPS. The pharmacy workforce, the third largest workforce group in the NHS, has in recent years had its potential to contribute to the delivery of public health services recognised [35]. The use of pharmacy technicians, trained as lifestyle coaches, in the delivery of DPS has particularly been identified as a viable option in terms of cost and availability in the USA [15]. With the NHS long term plan supporting the introduction of extended roles to ensure primary care networks can be more effective, pharmacy technicians could potentially be key players in the delivery of DPS [19]. As highlighted by this study, technicians delivering DPS would need multifaceted training including theory on pre-diabetes management, consultation, coaching and behaviour change skills.

### Strengths and limitations

This is the first study exploring the community pharmacy setting for delivering diabetes prevention services from the perspective of multiple stakeholders. It adds to an emerging body of research applying the COM-B model to assist theory informed approaches to developing diabetes prevention interventions [39]. The use of the COM-B model to identify barriers and facilitators, provides a theoretical basis for identifying suitable interventions and behaviour change techniques (through the Behaviour Change Wheel framework) that could enable the successful delivery of DPS in the community pharmacy setting. Further research is currently being undertaken to develop an intervention with strategies which will promote engagement and enable the successful delivery of DPS in the community pharmacy setting.

The barriers and facilitators identified by this research could be considered when designing other, non-diabetes related, interventions in the community pharmacy setting. In England, with the role of pharmacists and pharmacy technicians expanding beyond dispensing to the clinical management and prevention of chronic conditions such as cardiovascular disease, the findings of this research could facilitate the development of interventions promoting self-management in the community pharmacy setting [19, 40].

A limitation of the study was the lack of participants who are directly involved in commissioning the current NHS DPP. Additionally, the use of two different data collection methods, although useful for triangulation, generated two different types of data where interviews with general practice participants and commissioners generated in depth data whilst focus groups with community pharmacy participants generated superficial data. Arguably, more ground was covered with general practice participants than community pharmacy participants, thus inadvertently, this may have caused an imbalance in the data.

## Conclusions

This research highlights the potential for community pharmacy to increase accessibility of both screening and lifestyle interventions in primary care. New models of services should also seek to integrate community pharmacy services in primary care to facilitate efficient communication with general practices and promote better working relationships. To enhance the capability and motivation of community pharmacy to deliver such services, multifaceted training involving coaching and behaviour change skills and the appropriate use of pharmacy skill mix is required.
